# Health systems: the challenge of adapting and responding to the accelerating health transition in low income countries

**DOI:** 10.1186/1472-6963-14-S2-O4

**Published:** 2014-07-07

**Authors:** Don de Savigny

**Affiliations:** 1 Swiss Tropical and Public Health Institute, University of Basel, Basel, Switzerland

## 

In low-income countries, and particularly those in Africa, there are two important landscapes that have changed profoundly in recent years. One is the architecture of global health with its resulting increase in both the volume and complexity of health financing at country level. The second is the accelerating health transition in such countries where the past decade has witnessed declines in child mortality and increases in life expectancy at paces that have never been seen before in any period of history in any society. These health gains are not just a result of improving socio-economic development, but are consequent to important changes in health services and systems that have improved access to and coverage of several efficacious and cost effective essential health interventions funded in part by global health initiatives.

The dynamics of this health transition naturally result in very different and continually changing patterns of risk factors and attendant burdens of disease. Unfortunately the improvements in health systems in such countries have not yet included the necessary changes in, or even development of, appropriate means of monitoring the dynamics of the disease and risk factor patterns on a routine basis suitable for forward planning and policy making that will adequately steer development of the future health systems needed to respond to these dynamics. We remain too dependent on burden of disease modelling based on intelligent, but not sufficiently empirical real time data at country level on disease and risk factor dynamics. This is particularly so for cause of death data of acceptable coverage and quality in low-income countries where premature mortality still constitutes the largest share of disease burden DALYs. At the same time, the share of burden constituted by disability is rising rapidly from a much more varied set of causes and risks which are also changing due to demographic transition and other evolving phenomenon such as urbanization and globalization. These dynamics will require very different health systems and policies.

This presentation will discuss the need for linking three critical health information sources: 1) radical new approaches to routine longitudinal civil registration and vital statistics for disease burden monitoring, coupled with: 2) periodic national risk factor surveys that link to: 3) new approaches to monitoring district level health service coverage of services needed in response to the changing burdens and risks. Integrating across these data sources would provide the missing information strategy for evidence to feed more responsive health system planning and policy making needed for achieving universal health coverage.

